# 2-(3-Ethyl­sulfanyl-5-phenyl-1-benzofuran-2-yl)acetic acid

**DOI:** 10.1107/S160053680903253X

**Published:** 2009-08-22

**Authors:** Hong Dae Choi, Pil Ja Seo, Byeng Wha Son, Uk Lee

**Affiliations:** a Department of Chemistry, Dongeui University, San 24 Kaya-dong Busanjin-gu, Busan 614-714, Republic of Korea; b Department of Chemistry, Pukyong National University, 599-1 Daeyeon 3-dong, Nam-gu, Busan 608-737, Republic of Korea

## Abstract

The title compound, C_18_H_16_O_3_S, crystallizes with two symmetry-independent mol­ecules in the asymmetric unit. The phenyl rings are rotated out of the benzofuran planes, making dihedral angles of 43.38 (7) and 56.13 (6)° in the two mol­ecules. The carboxyl groups are involved in inversion-related inter­molecular O—H⋯O hydrogen bonds, which link the mol­ecules into centrosymmetric dimers. These dimers are further packed into stacks along the *b* axis by weak non-classical inter­molecular C—H⋯O hydrogen bonds. The crystal structure also exhibits inter­molecular C—H⋯π inter­actions, and two aromatic π–π inter­actions between the furan rings of neighbouring benzofuran systems; the centroid–centroid distances are 3.500 (3) and 3.605 (3) Å.

## Related literature

For the crystal structures of similar 2-(5-aryl-1-benzofuran-2-yl)acetic acid derivatives, see: Choi *et al.* (2007**a* ▶,b* ▶). For the pharmacological activity of benzofuran compounds, see: Howlett *et al.* (1999 ▶); Twyman & Allsop (1999 ▶).
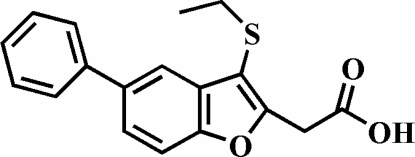


## Experimental

### 

#### Crystal data

C_18_H_16_O_3_S
*M* *_r_* = 312.37
Monoclinic, 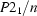

*a* = 12.4250 (7) Å
*b* = 11.7823 (7) Å
*c* = 21.2426 (13) Å
β = 93.021 (1)°
*V* = 3105.5 (3) Å^3^
*Z* = 8
Mo *K*α radiation
μ = 0.22 mm^−1^
*T* = 293 K
0.40 × 0.40 × 0.20 mm

#### Data collection

Bruker SMART CCD diffractometer
Absorption correction: multi-scan (*SADABS*; Sheldrick, 1996 ▶) *T*_min_ = 0.918, *T*_max_ = 0.958
18961 measured reflections
7033 independent reflections
4490 reflections with *I* > 2σ(*I*)
*R*_int_ = 0.051

#### Refinement

*R*[*F* ^2^ > 2σ(*F* ^2^)] = 0.044
*wR*(*F* ^2^) = 0.116
*S* = 1.02
7033 reflections
405 parameters
2 restraints
H atoms treated by a mixture of independent and constrained refinement
Δρ_max_ = 0.27 e Å^−3^
Δρ_min_ = −0.35 e Å^−3^

### 

Data collection: *SMART* (Bruker, 2007 ▶); cell refinement: *SAINT* (Bruker, 2007 ▶); data reduction: *SAINT*; program(s) used to solve structure: *SHELXS97* (Sheldrick, 2008 ▶); program(s) used to refine structure: *SHELXL97* (Sheldrick, 2008 ▶); molecular graphics: *ORTEP-3* (Farrugia, 1997 ▶) and *DIAMOND* (Brandenburg, 1999 ▶); software used to prepare material for publication: *SHELXL97*.

## Supplementary Material

Crystal structure: contains datablocks global, I. DOI: 10.1107/S160053680903253X/nk2002sup1.cif

Structure factors: contains datablocks I. DOI: 10.1107/S160053680903253X/nk2002Isup2.hkl

Additional supplementary materials:  crystallographic information; 3D view; checkCIF report

## Figures and Tables

**Table 1:** Hydrogen-bond geometry (Å, °)

*D*—H⋯*A*	*D*—H	H⋯*A*	*D*⋯*A*	*D*—H⋯*A*
O2—H2*O*⋯O3^i^	0.92 (2)	1.73 (2)	2.643 (2)	174 (3)
O5—H5*O*⋯O6^ii^	0.92 (2)	1.68 (2)	2.602 (2)	175 (4)
C6—H6⋯O5^iii^	0.93	2.57	3.461 (3)	161
C24—H24⋯O3	0.93	2.57	3.480 (3)	165
C12—H12⋯*Cg*4^iv^	0.93	2.91	3.561 (3)	131
C30—H30⋯*Cg*2^v^	0.93	2.80	3.506 (3)	133

Symmetry codes: (i) 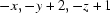; (ii) 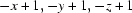; (iii) 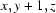; (iv) 
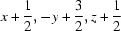
; (v) 
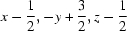
. *Cg*2 and *Cg*4 are the centroids of the C2–C7 and the C20–C25 benzene rings, respectively.

**Fig. 1.**
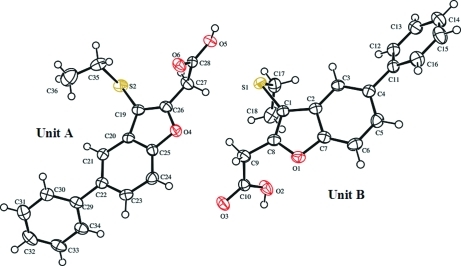
The molecular structure of the title compound with the atom numbering scheme. Displacement ellipsoids are drawn at the 50% probability level. H atoms are presented as a small cycles of arbitrary radius.

**Fig. 2.**
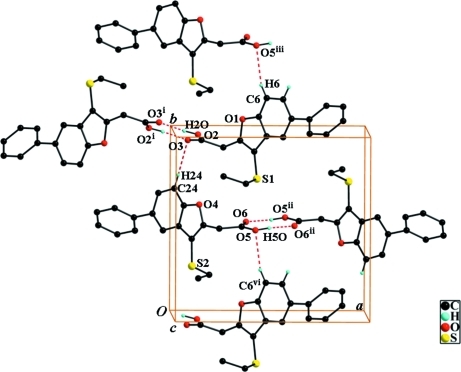
O–H···O and C–H···O hydrogen bonds (dotted lines) in the title compound. [Symmetry code: (i) - x, - y + 2, - z + 1; (ii) - x + 1, - y + 1, - z + 1; (iii) x, y + 1, z; (vi) x, y - 1, z.]

**Fig. 3.**
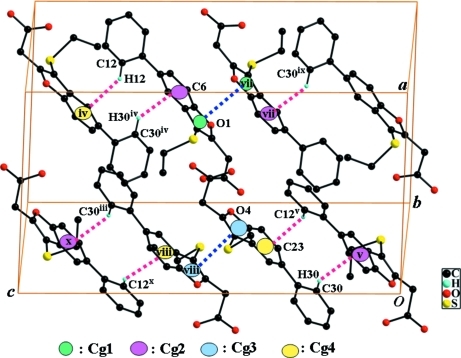
C–H···π and π–π interactions (dotted lines) in the title compound. Cg denotes the ring centroids. [Symmetry code: (iv) x + 1/2, - y + 3/2, z + 1/2; (v) x - 1/2, - y + 3/2, z - 1/2; (vii) -x + 1, -y + 2, -z + 1; (viii) - x, -y + 1, -z + 1; (ix) - x + 1/2, y + 1/2, -z + 1/2; (x) -x + 1/2, y - 1/2, -z + 3/2.]

## supplementary crystallographic information

### Comment

The benzofuran ring systems have attracted considerable interest in the view of their pharmacological properties (Howlett *et al.*, 1999; Twyman & Allsop, 1999). As a part of our ongoing studies on the synthesis and structures of 2-(5-aryl-1-benzofuran-2-yl)acetic acid analogues, the crystal structure of 2-(3-methylsulfanyl-5-phenyl-1-benzofuran-2-yl)acetic acid (Choi *et al.*, 2007*a*) and 2-[5-(4-bromophenyl)-3-methylsulfanyl-1-benzofuran-2-yl]acetic acid (Choi *et al.*, 2007*b*) have been described in the literature. Here we report the crystal structure of the title compound, which has two unique molecules in the asymmetric unit (further marked as A and B) (Fig. 1).

The benzofuran unit is essentially planar, with a mean deviation of 0.006 (2) Å for A, and 0.024 (2) Å for B, respectively, from the least-squares plane defined by the nine constituent atoms. In the title compound, the dihedral angles formed by the phenyl ring and the plane of the benzofuran fragment are 43.38 (7)  in A and 56.13 (6)° in B, respectively. In the crystal structure, the carboxylic acid groups are involved in inversion-related intermolecular O–H···O hydrogen bonds, which link the molecules into centrosymmetric dimers. These dimers are further packed into stacks along the *b* axis by non-classical intermolecular C–H···O hydrogen bonds (Table 1 and Fig. 2). The crystal structure (Fig. 3) is further stabilized by intermolecular C–H···π interactions between the phenyl H atom and the benzene ring of the adjacent molecule, with a C12–H12···Cg4^iv^ and a C30–H30···Cg2^v^ (Table 1; Cg2 and Cg4 are the centroids of the C2-C7 and the C20-C25 benzene rings, respectively). The crystal packing (Fig. 3) also shows two aromatic π–π interactions between the furan rings of the adjacent benzofuran molecules, with a Cg1···Cg1^vii^ and a Cg3···Cg3^viii^ distances of 3.500 (3) and 3.605 (3) Å, respectively (Cg1 and Cg3 are the centroids of the C1/C2/C7/O1/C8 and the C19/C20/C25/O4/C26 furan rings, respectively).

### Experimental

Ethyl 2-(3-ethylsulfanyl-5-phenyl-1-benzofuran-2-yl)acetate (374 mg, 1.1 mmol) was added to a solution of potassium hydroxide (309 mg, 5.5 mmol) in water (20 ml) and methanol (20 ml), and the mixture was refluxed for 6h, then cooled. Water was added, and the solution was extracted with dichloromethane. The aqueous layer was acidified to pH 1 with concentrated hydrochloric acid and then extracted with chloroform, dried over magnesium sulfate, filtered and concentrated under vacuum. The residue was purified by column chromatography (ethyl acetate) to afford the title compound as a colorless solid [yield 80%, m.p. 413-414 K; *R*_f_ = 0.79 (ethyl acetate)]. Single crystals suitable for X-ray diffraction were prepared by evaporation of a solution of the title compound in diisopropyl ether at room temperature.

### Refinement

Atoms H2O and H5O of the hydroxy groups was found in a difference Fourier map and were refined with an O–H distance restraint of 0.82 (3) Å. The other H atoms were positioned geometrically and refined using a riding model, with C–H = 0.93 Å for the aryl, 0.97 Å for the methylene, and 0.96 Å for the methyl H atoms with *U*_iso_(H) = 1.2*U*_eq_(C) for the aryl, methylene and methyl H atoms.

### Crystal data

**Table:**

C_18_H_16_O_3_S	*F*(000) = 1312
*M**_r_* = 312.37	*D*_x_ = 1.336 Mg m^−^^3^
Monoclinic, *P*2_1_/*n*	Melting point = 413–414 K
Hall symbol: -P 2yn	Mo *K*α radiation, λ = 0.71073 Å
*a* = 12.4250 (7) Å	Cell parameters from 5569 reflections
*b* = 11.7823 (7) Å	θ = 2.4–27.2°
*c* = 21.2426 (13) Å	µ = 0.22 mm^−^^1^
β = 93.021 (1)°	*T* = 293 K
*V* = 3105.5 (3) Å^3^	Block, colorless
*Z* = 8	0.40 × 0.40 × 0.20 mm

### Data collection

**Table:**

Bruker SMART CCD diffractometer	7033 independent reflections
Radiation source: fine-focus sealed tube	4490 reflections with *I* > 2σ(*I*)
graphite	*R*_int_ = 0.051
Detector resolution: 10.0 pixels mm^-1^	θ_max_ = 27.5°, θ_min_ = 1.9°
φ and ω scans	*h* = −16→14
Absorption correction: multi-scan (*SADABS*; Sheldrick, 1996)	*k* = −11→15
*T*_min_ = 0.918, *T*_max_ = 0.958	*l* = −22→27
18961 measured reflections	

### Refinement

**Table:**

Refinement on *F*^2^	Primary atom site location: structure-invariant direct methods
Least-squares matrix: full	Secondary atom site location: difference Fourier map
*R*[*F*^2^ > 2σ(*F*^2^)] = 0.044	Hydrogen site location: difference Fourier map
*wR*(*F*^2^) = 0.116	H atoms treated by a mixture of independent and constrained refinement
*S* = 1.01	*w* = 1/[σ^2^(*F*_o_^2^) + (0.0418*P*)^2^ + 1.3759*P*] where *P* = (*F*_o_^2^ + 2*F*_c_^2^)/3
7033 reflections	(Δ/σ)_max_ < 0.001
405 parameters	Δρ_max_ = 0.27 e Å^−^^3^
2 restraints	Δρ_min_ = −0.35 e Å^−^^3^

### Special details

**Table:**

Geometry. All esds (except the esd in the dihedral angle between two l.s. planes) are estimated using the full covariance matrix. The cell esds are taken into account individually in the estimation of esds in distances, angles and torsion angles; correlations between esds in cell parameters are only used when they are defined by crystal symmetry. An approximate (isotropic) treatment of cell esds is used for estimating esds involving l.s. planes.
Refinement. Refinement of F^2^ against ALL reflections. The weighted R-factor wR and goodness of fit S are based on F^2^, conventional R-factors R are based on F, with F set to zero for negative F^2^. The threshold expression of F^2^ > 2sigma(F^2^) is used only for calculating R-factors(gt) etc. and is not relevant to the choice of reflections for refinement. R-factors based on F^2^ are statistically about twice as large as those based on F, and R- factors based on ALL data will be even larger.

### Fractional atomic coordinates and isotropic or equivalent isotropic displacement parameters (Å^2^)

**Table:**

	*x*	*y*	*z*	*U*_iso_*/*U*_eq_	
S1	0.42821 (4)	0.74794 (5)	0.56883 (3)	0.03157 (15)	
S2	0.09978 (5)	0.26751 (5)	0.45920 (3)	0.03627 (16)	
O1	0.35424 (11)	1.06793 (13)	0.52955 (7)	0.0307 (3)	
O2	0.12491 (13)	0.98536 (16)	0.54306 (7)	0.0429 (4)	
H2O	0.054 (2)	1.005 (3)	0.5462 (16)	0.102 (13)*	
O3	0.07580 (12)	0.94865 (15)	0.44307 (7)	0.0366 (4)	
O4	0.13576 (11)	0.60092 (13)	0.46153 (7)	0.0316 (4)	
O5	0.42194 (12)	0.46922 (13)	0.56302 (7)	0.0314 (4)	
H5O	0.493 (2)	0.481 (3)	0.5539 (17)	0.116 (14)*	
O6	0.37647 (12)	0.50831 (14)	0.46214 (7)	0.0353 (4)	
C1	0.41378 (16)	0.89499 (18)	0.56241 (9)	0.0254 (5)	
C2	0.47608 (16)	0.98018 (18)	0.59689 (9)	0.0248 (5)	
C3	0.55970 (16)	0.97787 (18)	0.64336 (9)	0.0265 (5)	
H3	0.5871	0.9091	0.6585	0.032*	
C4	0.60141 (16)	1.08000 (18)	0.66661 (10)	0.0271 (5)	
C5	0.55883 (17)	1.18297 (19)	0.64317 (10)	0.0330 (5)	
H5	0.5874	1.2507	0.6591	0.040*	
C6	0.47558 (18)	1.1869 (2)	0.59704 (11)	0.0343 (5)	
H6	0.4477	1.2554	0.5816	0.041*	
C7	0.43644 (16)	1.08408 (19)	0.57530 (10)	0.0282 (5)	
C8	0.34212 (16)	0.95192 (19)	0.52408 (10)	0.0275 (5)	
C9	0.25729 (16)	0.9119 (2)	0.47742 (10)	0.0311 (5)	
H9A	0.2575	0.8295	0.4776	0.037*	
H9B	0.2764	0.9363	0.4358	0.037*	
C10	0.14441 (17)	0.95190 (18)	0.48781 (10)	0.0272 (5)	
C11	0.69182 (16)	1.07951 (19)	0.71553 (10)	0.0287 (5)	
C12	0.69211 (17)	1.0048 (2)	0.76654 (10)	0.0313 (5)	
H12	0.6349	0.9547	0.7702	0.038*	
C13	0.77631 (18)	1.0042 (2)	0.81172 (10)	0.0363 (6)	
H13	0.7749	0.9545	0.8457	0.044*	
C14	0.86258 (19)	1.0771 (2)	0.80665 (11)	0.0412 (6)	
H14	0.9195	1.0762	0.8369	0.049*	
C15	0.86381 (19)	1.1513 (2)	0.75639 (11)	0.0440 (6)	
H15	0.9218	1.2003	0.7528	0.053*	
C16	0.77908 (18)	1.1532 (2)	0.71117 (11)	0.0369 (6)	
H16	0.7804	1.2039	0.6777	0.044*	
C17	0.3695 (2)	0.7258 (2)	0.64445 (11)	0.0388 (6)	
H17A	0.3726	0.6456	0.6547	0.047*	
H17B	0.4122	0.7662	0.6768	0.047*	
C18	0.2544 (2)	0.7653 (2)	0.64531 (12)	0.0441 (6)	
H18A	0.2113	0.7246	0.6140	0.053*	
H18B	0.2509	0.8451	0.6363	0.053*	
H18C	0.2276	0.7513	0.6862	0.053*	
C19	0.09835 (16)	0.41459 (19)	0.44545 (10)	0.0272 (5)	
C20	0.03217 (15)	0.47991 (18)	0.40052 (9)	0.0249 (5)	
C21	−0.04696 (15)	0.45394 (18)	0.35359 (9)	0.0260 (5)	
H21	−0.0636	0.3789	0.3436	0.031*	
C22	−0.10028 (16)	0.54269 (19)	0.32209 (9)	0.0262 (5)	
C23	−0.07088 (17)	0.65529 (19)	0.33561 (10)	0.0300 (5)	
H23	−0.1065	0.7135	0.3135	0.036*	
C24	0.00971 (17)	0.68284 (19)	0.38098 (10)	0.0323 (5)	
H24	0.0298	0.7575	0.3894	0.039*	
C25	0.05787 (16)	0.59262 (19)	0.41258 (9)	0.0268 (5)	
C26	0.15723 (16)	0.4906 (2)	0.48009 (10)	0.0294 (5)	
C27	0.23707 (16)	0.4758 (2)	0.53380 (10)	0.0343 (5)	
H27A	0.2263	0.4018	0.5524	0.041*	
H27B	0.2232	0.5324	0.5655	0.041*	
C28	0.35276 (17)	0.48548 (18)	0.51715 (9)	0.0258 (5)	
C29	−0.19404 (16)	0.51884 (19)	0.27720 (10)	0.0279 (5)	
C30	−0.18684 (18)	0.44420 (19)	0.22683 (10)	0.0316 (5)	
H30	−0.1214	0.4094	0.2198	0.038*	
C31	−0.27602 (19)	0.4213 (2)	0.18717 (11)	0.0384 (6)	
H31	−0.2700	0.3727	0.1531	0.046*	
C32	−0.37435 (19)	0.4708 (2)	0.19823 (11)	0.0409 (6)	
H32	−0.4347	0.4541	0.1721	0.049*	
C33	−0.38292 (18)	0.5449 (2)	0.24798 (11)	0.0390 (6)	
H33	−0.4489	0.5783	0.2553	0.047*	
C34	−0.29342 (17)	0.5695 (2)	0.28696 (10)	0.0339 (5)	
H34	−0.2994	0.6203	0.3201	0.041*	
C35	0.1906 (2)	0.2174 (2)	0.40061 (12)	0.0438 (6)	
H35A	0.2462	0.2740	0.3956	0.053*	
H35B	0.2256	0.1485	0.4161	0.053*	
C36	0.1365 (3)	0.1937 (3)	0.33772 (13)	0.0640 (8)	
H36A	0.1025	0.2616	0.3216	0.077*	
H36B	0.0830	0.1357	0.3418	0.077*	
H36C	0.1890	0.1685	0.3093	0.077*	

### Atomic displacement parameters (Å^2^)

**Table:**

	*U*^11^	*U*^22^	*U*^33^	*U*^12^	*U*^13^	*U*^23^
S1	0.0307 (3)	0.0256 (3)	0.0383 (3)	0.0037 (2)	0.0005 (2)	−0.0017 (2)
S2	0.0380 (3)	0.0335 (3)	0.0378 (3)	0.0016 (3)	0.0059 (3)	0.0099 (3)
O1	0.0293 (8)	0.0282 (9)	0.0342 (8)	0.0021 (7)	−0.0007 (6)	0.0067 (7)
O2	0.0287 (9)	0.0706 (13)	0.0294 (9)	0.0054 (9)	−0.0003 (7)	−0.0133 (8)
O3	0.0310 (9)	0.0518 (11)	0.0262 (8)	0.0020 (8)	−0.0051 (7)	−0.0036 (7)
O4	0.0277 (8)	0.0346 (9)	0.0319 (8)	−0.0012 (7)	−0.0023 (6)	−0.0039 (7)
O5	0.0272 (8)	0.0363 (9)	0.0304 (8)	0.0015 (7)	−0.0027 (7)	0.0053 (7)
O6	0.0303 (8)	0.0525 (11)	0.0232 (8)	0.0004 (8)	0.0012 (6)	0.0001 (7)
C1	0.0229 (10)	0.0266 (11)	0.0271 (11)	0.0009 (9)	0.0043 (8)	0.0016 (9)
C2	0.0205 (10)	0.0260 (12)	0.0285 (11)	0.0001 (9)	0.0064 (8)	0.0032 (9)
C3	0.0228 (10)	0.0275 (12)	0.0294 (11)	0.0022 (9)	0.0046 (9)	0.0029 (9)
C4	0.0233 (10)	0.0299 (12)	0.0289 (11)	−0.0010 (9)	0.0072 (9)	−0.0011 (9)
C5	0.0308 (12)	0.0284 (12)	0.0400 (13)	−0.0057 (10)	0.0038 (10)	−0.0017 (10)
C6	0.0357 (13)	0.0258 (12)	0.0419 (13)	0.0027 (10)	0.0052 (10)	0.0066 (10)
C7	0.0241 (11)	0.0309 (12)	0.0296 (12)	−0.0004 (10)	0.0026 (9)	0.0042 (9)
C8	0.0241 (11)	0.0300 (12)	0.0288 (11)	0.0022 (9)	0.0049 (9)	0.0025 (9)
C9	0.0290 (11)	0.0367 (13)	0.0277 (12)	0.0031 (10)	0.0010 (9)	0.0009 (10)
C10	0.0298 (11)	0.0277 (12)	0.0241 (11)	−0.0016 (9)	−0.0003 (9)	0.0024 (9)
C11	0.0242 (11)	0.0325 (13)	0.0297 (12)	−0.0016 (9)	0.0045 (9)	−0.0065 (9)
C12	0.0266 (11)	0.0367 (13)	0.0311 (12)	−0.0024 (10)	0.0070 (9)	−0.0048 (10)
C13	0.0341 (13)	0.0451 (15)	0.0299 (12)	0.0029 (11)	0.0033 (10)	−0.0042 (11)
C14	0.0299 (12)	0.0603 (18)	0.0331 (13)	−0.0016 (12)	−0.0021 (10)	−0.0076 (12)
C15	0.0303 (13)	0.0567 (17)	0.0449 (15)	−0.0143 (12)	0.0022 (11)	−0.0062 (13)
C16	0.0329 (12)	0.0422 (15)	0.0361 (13)	−0.0069 (11)	0.0052 (10)	0.0010 (11)
C17	0.0535 (15)	0.0279 (13)	0.0345 (13)	−0.0050 (11)	−0.0033 (11)	0.0058 (10)
C18	0.0526 (16)	0.0373 (15)	0.0439 (14)	−0.0085 (12)	0.0185 (12)	0.0026 (11)
C19	0.0227 (10)	0.0312 (12)	0.0282 (11)	0.0026 (9)	0.0057 (9)	0.0036 (9)
C20	0.0191 (10)	0.0294 (12)	0.0268 (11)	0.0028 (9)	0.0061 (8)	0.0011 (9)
C21	0.0218 (10)	0.0270 (11)	0.0296 (11)	−0.0031 (9)	0.0063 (9)	−0.0010 (9)
C22	0.0226 (10)	0.0315 (12)	0.0249 (11)	0.0013 (9)	0.0056 (8)	0.0021 (9)
C23	0.0301 (11)	0.0278 (12)	0.0323 (12)	0.0050 (10)	0.0041 (9)	0.0052 (9)
C24	0.0312 (12)	0.0268 (12)	0.0388 (13)	−0.0014 (10)	0.0023 (10)	−0.0018 (10)
C25	0.0213 (10)	0.0332 (13)	0.0262 (11)	−0.0010 (9)	0.0028 (8)	−0.0027 (9)
C26	0.0228 (11)	0.0367 (13)	0.0289 (12)	0.0011 (10)	0.0038 (9)	0.0016 (10)
C27	0.0271 (11)	0.0487 (15)	0.0270 (12)	0.0003 (11)	0.0007 (9)	0.0008 (10)
C28	0.0280 (11)	0.0235 (11)	0.0255 (11)	0.0008 (9)	−0.0023 (9)	−0.0024 (9)
C29	0.0261 (11)	0.0312 (12)	0.0265 (11)	−0.0024 (9)	0.0024 (9)	0.0092 (9)
C30	0.0294 (12)	0.0332 (13)	0.0325 (12)	−0.0028 (10)	0.0049 (10)	0.0045 (10)
C31	0.0448 (14)	0.0383 (14)	0.0317 (13)	−0.0102 (12)	−0.0009 (11)	0.0041 (10)
C32	0.0331 (13)	0.0523 (17)	0.0364 (14)	−0.0140 (12)	−0.0085 (10)	0.0134 (12)
C33	0.0247 (12)	0.0546 (17)	0.0375 (14)	0.0010 (11)	0.0010 (10)	0.0135 (12)
C34	0.0301 (12)	0.0417 (14)	0.0301 (12)	0.0024 (11)	0.0028 (10)	0.0067 (10)
C35	0.0431 (14)	0.0326 (14)	0.0567 (16)	0.0088 (12)	0.0122 (12)	0.0058 (12)
C36	0.082 (2)	0.058 (2)	0.0526 (18)	0.0103 (17)	0.0174 (16)	−0.0032 (15)

### Geometric parameters (Å, °)

**Table:**

S1—C1	1.746 (2)	C16—H16	0.9300
S1—C17	1.818 (2)	C17—C18	1.505 (3)
S2—C19	1.757 (2)	C17—H17A	0.9700
S2—C35	1.822 (2)	C17—H17B	0.9700
O1—C8	1.379 (3)	C18—H18A	0.9600
O1—C7	1.385 (2)	C18—H18B	0.9600
O2—C10	1.273 (2)	C18—H18C	0.9600
O2—H2O	0.92 (2)	C19—C26	1.350 (3)
O3—C10	1.243 (2)	C19—C20	1.448 (3)
O4—C26	1.380 (3)	C20—C25	1.387 (3)
O4—C25	1.386 (2)	C20—C21	1.397 (3)
O5—C28	1.279 (2)	C21—C22	1.390 (3)
O5—H5O	0.92 (2)	C21—H21	0.9300
O6—C28	1.249 (2)	C22—C23	1.402 (3)
C1—C8	1.353 (3)	C22—C29	1.493 (3)
C1—C2	1.443 (3)	C23—C24	1.391 (3)
C2—C7	1.388 (3)	C23—H23	0.9300
C2—C3	1.395 (3)	C24—C25	1.377 (3)
C3—C4	1.390 (3)	C24—H24	0.9300
C3—H3	0.9300	C26—C27	1.482 (3)
C4—C5	1.404 (3)	C27—C28	1.503 (3)
C4—C11	1.490 (3)	C27—H27A	0.9700
C5—C6	1.388 (3)	C27—H27B	0.9700
C5—H5	0.9300	C29—C30	1.392 (3)
C6—C7	1.376 (3)	C29—C34	1.397 (3)
C6—H6	0.9300	C30—C31	1.383 (3)
C8—C9	1.485 (3)	C30—H30	0.9300
C9—C10	1.507 (3)	C31—C32	1.385 (3)
C9—H9A	0.9700	C31—H31	0.9300
C9—H9B	0.9700	C32—C33	1.379 (3)
C11—C16	1.395 (3)	C32—H32	0.9300
C11—C12	1.396 (3)	C33—C34	1.382 (3)
C12—C13	1.382 (3)	C33—H33	0.9300
C12—H12	0.9300	C34—H34	0.9300
C13—C14	1.382 (3)	C35—C36	1.490 (4)
C13—H13	0.9300	C35—H35A	0.9700
C14—C15	1.380 (4)	C35—H35B	0.9700
C14—H14	0.9300	C36—H36A	0.9600
C15—C16	1.387 (3)	C36—H36B	0.9600
C15—H15	0.9300	C36—H36C	0.9600
			
C1—S1—C17	99.60 (10)	C17—C18—H18C	109.5
C19—S2—C35	101.98 (11)	H18A—C18—H18C	109.5
C8—O1—C7	105.57 (16)	H18B—C18—H18C	109.5
C10—O2—H2O	112 (2)	C26—C19—C20	106.23 (19)
C26—O4—C25	105.34 (16)	C26—C19—S2	124.28 (17)
C28—O5—H5O	115 (2)	C20—C19—S2	129.35 (16)
C8—C1—C2	106.20 (19)	C25—C20—C21	119.23 (19)
C8—C1—S1	126.92 (17)	C25—C20—C19	105.66 (18)
C2—C1—S1	126.88 (16)	C21—C20—C19	135.1 (2)
C7—C2—C3	119.26 (19)	C22—C21—C20	118.6 (2)
C7—C2—C1	105.91 (18)	C22—C21—H21	120.7
C3—C2—C1	134.8 (2)	C20—C21—H21	120.7
C4—C3—C2	119.0 (2)	C21—C22—C23	120.04 (19)
C4—C3—H3	120.5	C21—C22—C29	120.12 (19)
C2—C3—H3	120.5	C23—C22—C29	119.69 (19)
C3—C4—C5	119.69 (19)	C24—C23—C22	122.2 (2)
C3—C4—C11	119.8 (2)	C24—C23—H23	118.9
C5—C4—C11	120.5 (2)	C22—C23—H23	118.9
C6—C5—C4	122.2 (2)	C25—C24—C23	115.9 (2)
C6—C5—H5	118.9	C25—C24—H24	122.1
C4—C5—H5	118.9	C23—C24—H24	122.1
C7—C6—C5	116.4 (2)	C24—C25—O4	125.42 (19)
C7—C6—H6	121.8	C24—C25—C20	124.01 (19)
C5—C6—H6	121.8	O4—C25—C20	110.56 (18)
C6—C7—O1	126.2 (2)	C19—C26—O4	112.21 (18)
C6—C7—C2	123.5 (2)	C19—C26—C27	131.6 (2)
O1—C7—C2	110.25 (18)	O4—C26—C27	116.21 (19)
C1—C8—O1	112.04 (19)	C26—C27—C28	114.82 (18)
C1—C8—C9	131.7 (2)	C26—C27—H27A	108.6
O1—C8—C9	116.21 (18)	C28—C27—H27A	108.6
C8—C9—C10	115.85 (18)	C26—C27—H27B	108.6
C8—C9—H9A	108.3	C28—C27—H27B	108.6
C10—C9—H9A	108.3	H27A—C27—H27B	107.5
C8—C9—H9B	108.3	O6—C28—O5	124.23 (19)
C10—C9—H9B	108.3	O6—C28—C27	120.81 (18)
H9A—C9—H9B	107.4	O5—C28—C27	114.97 (18)
O3—C10—O2	123.9 (2)	C30—C29—C34	118.5 (2)
O3—C10—C9	118.97 (18)	C30—C29—C22	122.04 (19)
O2—C10—C9	117.10 (18)	C34—C29—C22	119.4 (2)
C16—C11—C12	118.3 (2)	C31—C30—C29	120.7 (2)
C16—C11—C4	120.7 (2)	C31—C30—H30	119.7
C12—C11—C4	120.99 (19)	C29—C30—H30	119.7
C13—C12—C11	120.9 (2)	C30—C31—C32	120.0 (2)
C13—C12—H12	119.6	C30—C31—H31	120.0
C11—C12—H12	119.6	C32—C31—H31	120.0
C12—C13—C14	120.3 (2)	C33—C32—C31	120.1 (2)
C12—C13—H13	119.9	C33—C32—H32	120.0
C14—C13—H13	119.9	C31—C32—H32	120.0
C15—C14—C13	119.6 (2)	C32—C33—C34	120.0 (2)
C15—C14—H14	120.2	C32—C33—H33	120.0
C13—C14—H14	120.2	C34—C33—H33	120.0
C14—C15—C16	120.4 (2)	C33—C34—C29	120.8 (2)
C14—C15—H15	119.8	C33—C34—H34	119.6
C16—C15—H15	119.8	C29—C34—H34	119.6
C15—C16—C11	120.5 (2)	C36—C35—S2	114.02 (19)
C15—C16—H16	119.7	C36—C35—H35A	108.7
C11—C16—H16	119.7	S2—C35—H35A	108.7
C18—C17—S1	113.11 (16)	C36—C35—H35B	108.7
C18—C17—H17A	109.0	S2—C35—H35B	108.7
S1—C17—H17A	109.0	H35A—C35—H35B	107.6
C18—C17—H17B	109.0	C35—C36—H36A	109.5
S1—C17—H17B	109.0	C35—C36—H36B	109.5
H17A—C17—H17B	107.8	H36A—C36—H36B	109.5
C17—C18—H18A	109.5	C35—C36—H36C	109.5
C17—C18—H18B	109.5	H36A—C36—H36C	109.5
H18A—C18—H18B	109.5	H36B—C36—H36C	109.5
			
C17—S1—C1—C8	−106.3 (2)	C35—S2—C19—C26	−99.5 (2)
C17—S1—C1—C2	73.8 (2)	C35—S2—C19—C20	85.5 (2)
C8—C1—C2—C7	−0.7 (2)	C26—C19—C20—C25	−0.1 (2)
S1—C1—C2—C7	179.23 (16)	S2—C19—C20—C25	175.61 (16)
C8—C1—C2—C3	178.9 (2)	C26—C19—C20—C21	−177.7 (2)
S1—C1—C2—C3	−1.2 (3)	S2—C19—C20—C21	−2.1 (4)
C7—C2—C3—C4	−0.3 (3)	C25—C20—C21—C22	−2.4 (3)
C1—C2—C3—C4	−179.8 (2)	C19—C20—C21—C22	175.0 (2)
C2—C3—C4—C5	0.1 (3)	C20—C21—C22—C23	2.9 (3)
C2—C3—C4—C11	−179.07 (18)	C20—C21—C22—C29	−172.70 (18)
C3—C4—C5—C6	0.0 (3)	C21—C22—C23—C24	−1.2 (3)
C11—C4—C5—C6	179.2 (2)	C29—C22—C23—C24	174.40 (19)
C4—C5—C6—C7	0.1 (3)	C22—C23—C24—C25	−1.0 (3)
C5—C6—C7—O1	−179.84 (19)	C23—C24—C25—O4	−176.59 (18)
C5—C6—C7—C2	−0.3 (3)	C23—C24—C25—C20	1.5 (3)
C8—O1—C7—C6	−179.2 (2)	C26—O4—C25—C24	177.8 (2)
C8—O1—C7—C2	1.2 (2)	C26—O4—C25—C20	−0.6 (2)
C3—C2—C7—C6	0.4 (3)	C21—C20—C25—C24	0.2 (3)
C1—C2—C7—C6	−180.0 (2)	C19—C20—C25—C24	−177.96 (19)
C3—C2—C7—O1	180.00 (17)	C21—C20—C25—O4	178.51 (17)
C1—C2—C7—O1	−0.3 (2)	C19—C20—C25—O4	0.4 (2)
C2—C1—C8—O1	1.5 (2)	C20—C19—C26—O4	−0.3 (2)
S1—C1—C8—O1	−178.41 (14)	S2—C19—C26—O4	−176.24 (14)
C2—C1—C8—C9	179.7 (2)	C20—C19—C26—C27	178.4 (2)
S1—C1—C8—C9	−0.2 (3)	S2—C19—C26—C27	2.5 (3)
C7—O1—C8—C1	−1.7 (2)	C25—O4—C26—C19	0.5 (2)
C7—O1—C8—C9	179.80 (17)	C25—O4—C26—C27	−178.42 (17)
C1—C8—C9—C10	122.9 (2)	C19—C26—C27—C28	102.0 (3)
O1—C8—C9—C10	−58.9 (3)	O4—C26—C27—C28	−79.3 (2)
C8—C9—C10—O3	160.6 (2)	C26—C27—C28—O6	2.4 (3)
C8—C9—C10—O2	−20.7 (3)	C26—C27—C28—O5	−177.9 (2)
C3—C4—C11—C16	136.0 (2)	C21—C22—C29—C30	−55.1 (3)
C5—C4—C11—C16	−43.2 (3)	C23—C22—C29—C30	129.3 (2)
C3—C4—C11—C12	−43.3 (3)	C21—C22—C29—C34	122.6 (2)
C5—C4—C11—C12	137.5 (2)	C23—C22—C29—C34	−53.0 (3)
C16—C11—C12—C13	0.4 (3)	C34—C29—C30—C31	0.4 (3)
C4—C11—C12—C13	179.7 (2)	C22—C29—C30—C31	178.1 (2)
C11—C12—C13—C14	−0.8 (3)	C29—C30—C31—C32	−1.5 (3)
C12—C13—C14—C15	0.5 (4)	C30—C31—C32—C33	1.4 (3)
C13—C14—C15—C16	0.1 (4)	C31—C32—C33—C34	−0.2 (4)
C14—C15—C16—C11	−0.5 (4)	C32—C33—C34—C29	−0.9 (3)
C12—C11—C16—C15	0.3 (3)	C30—C29—C34—C33	0.8 (3)
C4—C11—C16—C15	−179.1 (2)	C22—C29—C34—C33	−176.9 (2)
C1—S1—C17—C18	59.18 (19)	C19—S2—C35—C36	−86.9 (2)

### Hydrogen-bond geometry (Å, °)

**Table:**

*D*—H···*A*	*D*—H	H···*A*	*D*···*A*	*D*—H···*A*
O2—H2O···O3^i^	0.92 (2)	1.73 (2)	2.643 (2)	174 (3)
O5—H5O···O6^ii^	0.92 (2)	1.68 (2)	2.602 (2)	175 (4)
C6—H6···O5^iii^	0.93	2.57	3.461 (3)	161
C24—H24···O3	0.93	2.57	3.480 (3)	165
C12—H12···Cg4^iv^	0.93	2.91	3.561 (3)	131
C30—H30···Cg2^v^	0.93	2.80	3.506 (3)	133

Symmetry codes: (i) −*x*, −*y*+2, −*z*+1; (ii) −*x*+1, −*y*+1, −*z*+1; (iii) *x*, *y*+1, *z*; (iv) *x*+1/2, −*y*+3/2, *z*+1/2; (v) *x*−1/2, −*y*+3/2, *z*−1/2.
